# Elevation-dependent landsliding driven by climate change in the eastern Himalayan syntaxis

**DOI:** 10.1093/nsr/nwag238

**Published:** 2026-04-24

**Authors:** Chengbin Zou, John D Jansen, Xiangyang Dou, Lanxin Dai, Qiang Xu, Xuanmei Fan

**Affiliations:** State Key Laboratory of Geohazard Prevention and Geoenvironment Protection, Chengdu University of Technology, Chengdu 610059, China; GFÚ Institute of Geophysics, Czech Academy of Sciences, 141 00 Prague 4, Czechia; State Key Laboratory of Geohazard Prevention and Geoenvironment Protection, Chengdu University of Technology, Chengdu 610059, China; Faculty of Geo-Information Science and Earth Observation, University of Twente, 7522 NH Enschede, The Netherlands; State Key Laboratory of Geohazard Prevention and Geoenvironment Protection, Chengdu University of Technology, Chengdu 610059, China; State Key Laboratory of Geohazard Prevention and Geoenvironment Protection, Chengdu University of Technology, Chengdu 610059, China; State Key Laboratory of Geohazard Prevention and Geoenvironment Protection, Chengdu University of Technology, Chengdu 610059, China

**Keywords:** landslides, cryosphere, denudation rate, global warming

## Abstract

Has climate change escalated landsliding in high mountain regions? We compiled an inventory of 8607 climate-related landslides (1987–2020) in the eastern Himalayan syntaxis to investigate how hillslopes in high mountains, particularly cryospheric landscapes, are responding to recent trends in precipitation and temperature. We identify a 5-fold increase in annual landslide volumes at high elevations (>3000 m above sea level) over the past three decades. The landslide distribution is observed migrating upslope in the wake of retreating glaciers and melting permafrost—a trend underpinned by a warming-induced shift in precipitation falling as rain rather than snow. Today, climate-related landsliding in the eastern Himalayas is driving an extreme denudation rate of 1.0 ± 0.3 mm yr^–1^, comparable to that of alpine glaciation. Our findings imply that landslide activity across High Mountain Asia is evolving rapidly under climate change, amplifying the sediment cascade and heightening the risk of cascading mountain hazards.

## INTRODUCTION

Under contemporary climate change, the cryosphere is a nexus of transforming landscapes. Shifting patterns of precipitation and temperature are amplified in these cold, high elevation/latitude regions like nowhere else on Earth [1,2], increasing rates of erosion and sediment flux especially in landslide-dominant topography [3,4]. Hillslope instability ascribed to the direct or indirect effects of climate change has in turn triggered more frequent multi-hazard chains, laying waste to life and infrastructure across mountainous regions of the world [5,6]. As for the long-term implications of a transforming cryosphere, many questions remain unresolved: Is enhanced landsliding a recurring hallmark of mountain deglaciation? And how do rates of mass-wasting compare to other geomorphic agents, such as glaciers, in terms of mountain denudation?

Fundamentally, landslides occur when resistive forces within hillslopes are exceeded by driving forces under the influence of precipitation, temperature, seismicity, or human activities. Cryosphere landscapes are especially sensitive to climate-driven responses directly linked with precipitation and temperature, such as accelerated glacier retreat and permafrost thawing [7,8]. The first response exposes hillslopes and creates opportunities for landsliding, and thawing reduces their mechanical strength. All such processes share a marked dependence on elevation. Regions dominated by cryospheric processes have experienced elevation-dependent shifts in patterns of precipitation and temperature over recent decades [9]. Hence, it follows that shifts in the size and/or frequency of climate-related landslides may be detectable at differing elevations—a testable hypothesis with important implications over both short and long timescales.

### Has climate change intensified landsliding in the eastern Himalayan syntaxis?

**Figure 1.**
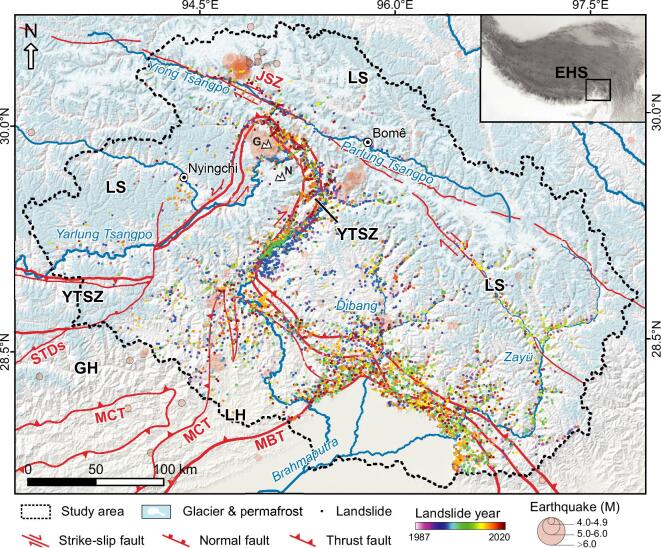
Geological and climatic setting of the EHS. Study area (94 706 km^2^) main structural divisions, seismic activity (1987–2020), landslides distribution (*n* = 8607), glaciers [65] and permafrost [19]. Major peaks are Namche Barwa (N) and Gyala Peri (G). Catchments include the Yiong/Parlung Tsangpo, Dibang and Zayü, plus the Yarlung Tsangpo lower reaches to the Brahmaputra. The EHS comprises three major tectono-stratigraphic units: (1) the Lhasa block (LS)—Precambrian metamorphics, Palaeozoic, and Mesozoic cover intruded by Mesozoic and Cenozoic plutons [87]; (2) the Yarlung–Tsangpo suture zone (YTSZ)—highly deformed and metamorphosed ophiolitic mélange [13,88]; and (3) the Himalayan sequences [13,87,89] subdivided by the Main Central Thrust (MCT) into the Greater Himalaya (GH) Precambrian and Neoproterozoic–Cambrian metamorphics, and the Lesser Himalaya (LH) low-grade metamorphics of Neoproterozoic–Cambrian and Palaeozoic age [13,90]. The Main Boundary Thrust (MBT) separates the Himalayan sequences from the Indian foreland basins. Two other major fault zones are the Jiali suture zone (JSZ) and Southern Tibet Detachment system (STDs). More detailed information on lithology and faults is shown in Fig. S5. The inset panel shows the geographic context of the EHS.

The Himalayan mountain belt is a global hotspot for landsliding [10,11] that is also sensitive to the effects of climate change [12]. Located at the frontier of the Indian–Asian collision, the eastern Himalayan syntaxis (EHS) is among the most geomorphically active regions on Earth due to the conjunction of rapid rock uplift, strong seismicity, and climatic factors [13–16]. At the core of the EHS, the Yarlung Tsangpo has carved 6 km of relief separating the >7 km peaks of Namche Barwa and Gyala Peri [17] (Fig. 1). The extreme relief promotes climatic zonation (Fig. S1) with mean annual temperatures spanning ∼20 to <0°C, while intense tropical moisture from two summer monsoons (the Indian and East Asian) injects up to 4000 mm of annual precipitation, declining from south to north [18]. The low temperatures and abundant precipitation have fostered extensive ice fields and permafrost at high elevations across the EHS [19] (Fig. 1). Over recent decades, however, mean annual temperatures in the EHS have risen, while annual precipitation shows a slight decrease (Fig. S2). Notably, the precipitation changes at higher elevations are not significant, and while warming has occurred at all elevations, high elevations have warmed at a faster rate (Fig. S1).

The effects of climate change in the EHS can be seen in notable glacier retreat [20,21], permafrost degradation [22,23], and the recent series of potentially related multi-hazard chains [24,25]. Further, the region is host to large-scale infrastructure projects, such as the Sichuan–Tibet railway [26], which have swelled the human population and heightened the risk of geological hazards [5,6]. Has climate change amplified rates of denudation and sediment flux across High Asia by boosting the magnitude and/or frequency of landsliding? Previous work records an upsurge in hillslope instability across several cryosphere regions of the world [27–30]; however, comprehensive testing of these ideas in High Mountain Asia requires reliable, long-term records [31,32] collected systematically across large spatial scales. Few such comprehensive analyses have been conducted to date.

In this study, we compiled a large inventory of climate-related landslides in the EHS at annual resolution (1987–2020) based on manual interpretation of satellite imagery (and excluding landslides linked to non-climate-related factors: earthquakes, mega outburst floods, and human activities). We analyze the inventory with the aim of detecting spatial and temporal patterns of landsliding, specifically testing for an elevation dependence tied to coeval trends in precipitation and temperature. Our results provide new insights to the impact of climate change on landslides in the EHS. We reflect on the implications of these findings over both short and long timescales for risk management and long-term landscape evolution of glacierized mountain belts.

## RESULTS

### Landslide distribution relative to topography, geology, and climate

**Figure 2.**
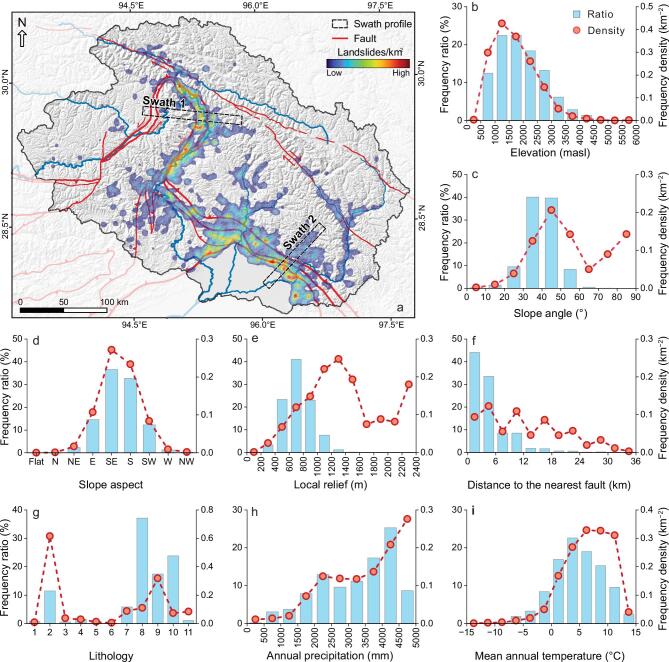
Landslide density map and eight factors governing landslide frequency. (a) Density map (*n* = 8607); (b) frequency vs. elevation; (c) frequency vs. slope angle; (d) frequency vs. slope aspect; (e) frequency vs. local relief; (f) frequency vs. distance to the nearest fault; (g) frequency vs. lithological group; (h) frequency vs. annual precipitation; and (i) frequency vs. mean annual temperature. Histograms show landslide numbers (% of total); dash lines show the frequency density (landslides/km^2^). More detailed information on lithology and faults is shown in Fig. S5. Lithological groups are 1—Quaternary, 2—Neogene sandstone, mudstone, and conglomerate, 3—Mesozoic sandstone, conglomerate, and siltstone, 4—Palaeozoic metamorphic sandstone, slate, carbonates, 5—Palaeozoic slate interbedded with limestone, sandstone, and volcanics, 6—Palaeozoic limestone and dolomite, 7—Neoproterozoic–Cambrian schist, slate, metamorphic sandstone, phyllite, 8—Precambrian gneiss, 9—Mesozoic ophiolitic mélange, 10—Granite, and 11—Undifferentiated.

Our 1987–2020 inventory contains a total of 8607 landslides after excluding those triggered by non-climate-related factors (Fig. 1 and Fig. S3). The largest single landslide in our inventory is ∼2.0 × 10^6^ m^2^ and 61.5% of landslides fall within 10^4^–10^5^ m^2^ (Table S1). For landslides >∼5.0 × 10^4^ m^2^, the probability density follows a power-law with an exponent (*β*) of 2.565 ± 0.074 (Fig. S4a). Our mapping shows that landslides tend to cluster around Yarlung Tsangpo gorge and the mountain valleys adjoining the southern rangefront (Figs 1, 2a and Fig. S3). To understand what influences this spatial distribution, we analyzed topographic, geological, and climatic factors, as follows.

The majority of landslides (77%) occur at low-medium elevations (1000–3000 masl) (Fig. 2b); around 80% correspond to hillslope angles of 30°–50° (Fig. 2c), and they show a clear preference (69%) for southern to southeastern slopes (Fig. 2d). Local relief of 400–1000 m concentrates 88% of all landslides (Fig. 2e).

Lithology and faults appear influential for landslide distribution (Fig. 2f, g). The frequency of landslides decreases rapidly with distance from faults: 44% of landslides occur within 3 km (Fig. 2f). The density of landslides is highest in the Neogene sandstone, mudstone, and conglomerate group (62%) followed by the Mesozoic ophiolitic mélange group (32%) (Fig. 2g and Fig. S5).

Precipitation appears to be the most important climate factor controlling the spatial distribution of landslides, although as we discuss below its influence is closely aligned with temperature. The frequency density of landslides is positively correlated with precipitation and 85% occur in areas with mean annual precipitation >2000 mm (Fig. 2h). Analysis of temperature (Fig. 2i) shows that the majority of landslides occur where mean annual temperature is >0°C, with just 14% falling in areas <0°C.

### Landslide intensity through time

**Figure 3.**
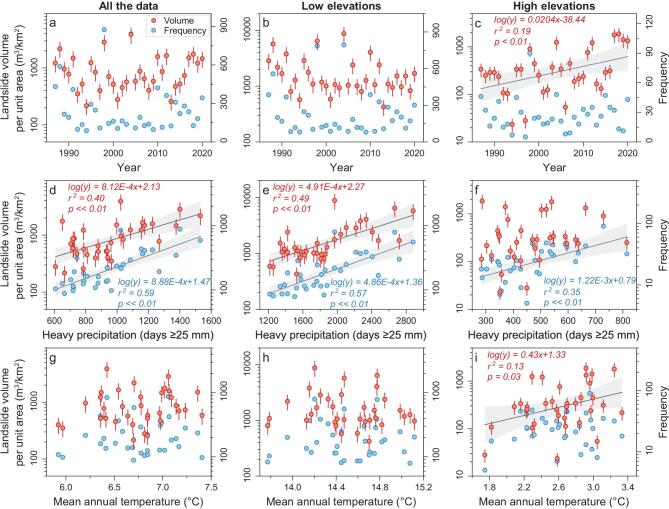
Historical trends and relationships between landslide volume, frequency, and climate factors at low (<3000 masl) and high (>3000 masl) elevations. (a–c) Landslide volume and frequency variations, 1987–2020. Annual volumes of high-elevation landslides have increased by ∼5-fold on average. (d–f) Landslide volume and frequency vs. heavy precipitation, which is defined as annual cumulative precipitation counting only days ≥25 mm. (g–i) Landslide volume and frequency vs. mean annual temperature. Note the logarithmic axes; linear regressions and 95% confidence intervals are fitted where appropriate. These relationships are treated with binary regression in Fig. S7. Using different scaling constants for volume estimation, the landslide trend remains almost unchanged (Fig. S14).

Landsliding intensity is a function of the frequency and total volume of landslides per year and fluctuates widely over the 34-year record (Fig. 3a–c and Table S2). Landslides are estimated to mobilize an average of 95.97 ± 29.2 million m^3^ of sediment per year (see Methods for our area–volume scaling calculations). There are no statistically significant historical trends in overall intensity of landsliding (Fig. 3a), but differences exist relative to elevation (Fig. S6). At high elevations (> 3000 masl), we detect a statistically significant increase in annual volumes (Fig. 3c); annual frequency appears steady at all elevations (Fig. 3a–c).

### Landslide size–frequency distribution

**Figure 4.**
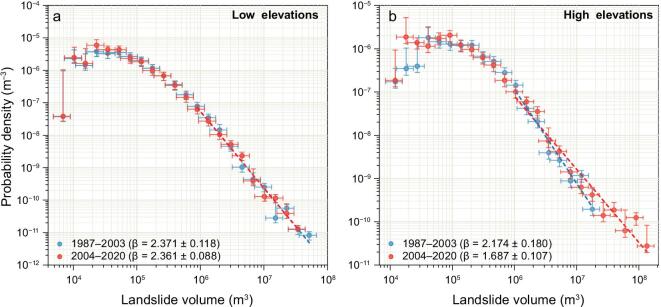
Landslide size-frequency distributions during split time-intervals for low (a) and high (b) elevations. These data are also shown in terms of landslide area (Fig. S4).

The size–frequency distribution of landslides is influenced by environmental change [33]. To investigate whether landslide size–frequency distribution has changed over time, the inventory was evenly subdivided into two periods: 1987–2003 and 2004–2020 (Fig. 4 and Fig. S4b, c). In each case, a power-law is a good fit for landslides with volumes >∼8 × 10^5^ m^3^. For low elevations, the power-law exponent (*β*) is consistent across the two time-intervals (Fig. 4a), indicating that the probability of large-scale landslides has remained steady through time, whereas *β* shows a statistically significant decrease at high elevations (Fig. 4b), suggesting enhanced probability of large-scale landslides (especially those >10^7^ m^3^). Landslide area-frequency distribution shows similar results (Fig. S4b, c).

### Landslide intensity and climate

Heavy precipitation (that is, annual cumulative precipitation for days ≥25 mm) and mean annual temperature were examined to explore the relationship between landslides and climate (Fig. 3 and Fig. S7). Note that analyses employing alternative parameters of precipitation and temperature (presented in Table S3) do not have any bearing on our findings. Landslide frequency correlates with heavy precipitation at all elevations (Fig. 3d–f), but not with mean temperature (Fig. 3g–i). At low elevations, landslide volumes exhibit significant correlations with heavy precipitation (Fig. 3e), but with temperature there is also no trend (Fig. 3h). At high elevations the relationship appears more complex. While precipitation clearly influences landslide frequency (Fig. 3e, f), landslide volumes relate only weakly to precipitation and temperature when these factors are considered separately (Fig. 3f, i). Combining the influence of precipitation and temperature in a binary regression analysis yields a stronger relationship with landslide volumes (Fig. S7).

## DISCUSSION

### Role of topography, lithology, faults, and climate on landsliding

The spatial distribution of landslides in the EHS is strongly controlled by the highly dissected topography. The majority of landslides occur at low-medium elevations 1000–3000 masl (Fig. 2b) and lie concentrated within deep valleys flanked by steep hillslopes (>30°) that are likely to be close to their failure threshold (Figs 1 and 2c) [34]. The topography itself is influenced by the system of regional faults and especially the densely fractured conjunction of the Indian and Eurasian plates known as the Yarlung–Tsangpo suture zone (Fig. 1). By reducing the mechanical strength of hillslopes, faults do unquestionably promote landsliding [35,36]; for instance, 91% of the landslides in our inventory occur within 10 km of a fault. Moreover, lithology, too, plays a role where major block faults create sharp contrasts in rock strength: the highest density of landslides occurs along the weak ophiolitic mélange rocks of the Yarlung–Tsangpo suture zone. And yet, it is important to note that the deeply incised drainage network that imposes an antecedent control on landsliding [34,37].

**Figure 5.**
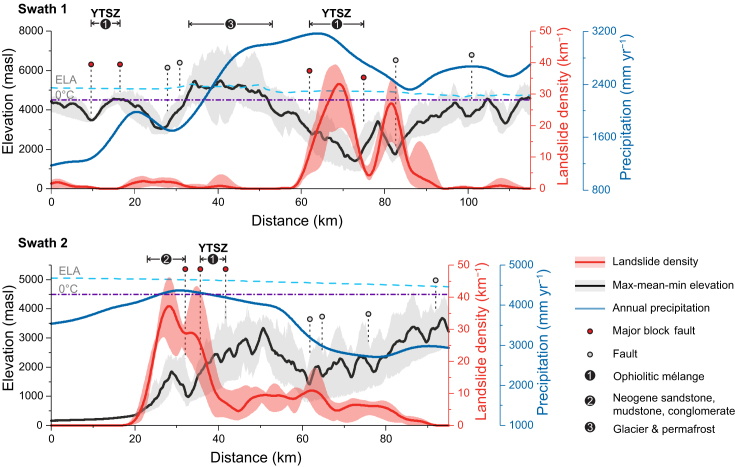
Swath profiles showing relationships between landslides, topography, geological, and climatic factors. East-to-west swath profile locations are indicated in Fig. 2a and Fig. S5. Shown are the mean elevation of the 0°C isotherm (dash-dot line) [63], and the average equilibrium line altitude (ELA, dash line) extracted from Fig. S15. The highly fractured Yarlung–Tsangpo suture zone locates the major valleys, as do the weak mélange and Neogene lithologies.

These effects and relationships are illustrated in two swaths (Fig. 5) constructed across the most prominent landslide clusters in the EHS. High landslide densities in swath 1 occur along the deeply incised Tsangpo gorge (and its tributary), corresponding to high precipitation (∼2400–3200 mm yr^–1^) and the weak ophiolitic rocks of the Yarlung–Tsangpo suture zone. Notable (though lower) landslide densities also occur where the topography draws close to or intersects the 0°C isotherm, suggesting that temperature-related cryospheric processes, may also modulate landslide occurrence. The rangefront area spanned by swath 2 hosts even higher landslide densities and emphasizes the functional relationships between extreme monsoon-driven precipitation (> 4000 mm yr^–1^), weak Neogene rocks and suture zone ophiolites tracing the deepest river valleys.

The governing effect of deeply dissected topography on the distribution of low-lying landslides is observed also in the Himalayan Karakorum Ranges by Blöthe *et al.* [37] who argue that landslides preferentially undermine high-relief landscapes from below in response to intense fluvial and glacial incision. Moreover, they identify the importance of vertically stacked erosional domains in the landscape: glacial and periglacial processes dominating high elevations transitioning to debris flows and fluvial processes below. We build on those observations here by targeting specifically landslides potentially associated with elevation-dependent shifts in patterns of precipitation and temperature that have occurred over recent decades in the EHS [22,38].

### Probability of large landslides has increased at high elevations

Mean annual temperatures in the EHS have warmed significantly at a rate of 0.23 ± 0.03°C decade^–1^, while annual precipitation shows a slight decreasing trend with time (Fig. S2). Has climate change intensified landsliding in the EHS? Our historical analysis sets out to test the hypothesis that changes in the magnitude and/or frequency of landslides in the EHS are detectable at differing elevations. We discover that the annual landslide volume has increased for elevations >3000 masl (Fig. 3c), and that this trend is a function mainly of several large-volume events over recent years (Fig. 4). Similar results were observed in other high mountain regions such as the Pamir Plateau [29,30], the western Tibetan Plateau [28], and the St Elias Mountains, Alaska [27]. The increasing trend is likely due to both temperature and precipitation drivers, as discussed below.

**Figure 6.**
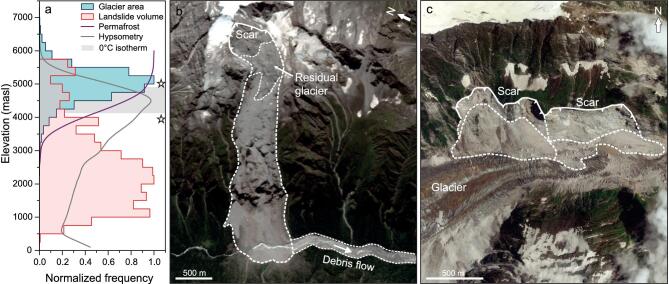
Landsliding in the cryosphere. (a) Elevation relationships between landslide volumes, the distribution of glaciers [65], and permafrost [19] (fractional area per elevation bin), the 0°C isotherm (interquartile-range calculated for 1979–2022, after ref. [63]), and hypsometry. Given the hypsometric maximum in surface area at ∼ 4000–5000 masl, even a modest rise in the 0°C isotherm or ELA increases the potential for new landslides. Upper–lower stars indicate headwall elevations of landslides in (b) and (c), respectively. (b) Landslide induced possibly by permafrost degradation/freeze-thaw intensification (Planet image, 6 August 2020, Fig. S15). (c) Landslide related to glacier retreat (Jilin-1 image, 6 July 2022, Fig. S15).

At high elevations, landslides, permafrost, and glaciers intersect (Fig. 6), and the cryosphere is undergoing rapid and profound changes associated with warming—in particular, glacier retreat [20,21], permafrost thaw [39] and reduction in solid precipitation [38,40,41]. Around 58% of areas >3000 masl are covered by glaciers and permafrost (Fig. 1). Since the end of the 19th century, valley and cirque glaciers in the EHS region have contracted in length by 24% and 28%, respectively, and the equilibrium line altitude (ELA) has risen 136 m [38]. By exposing new ground to solar radiation, glacier retreat has opened the way for permafrost degradation [42,43], such that over the past several decades the area of permafrost has shrunk by 28%, while the elevation of the 0°C isotherm has risen by order ∼100 m (Fig. S8).

Rising temperatures (Fig. S2b) have introduced four key factors pertinent to landslide activity in the EHS landscape: (i) retreating glaciers expose hillslopes and create opportunities for potential new landslides. Glacier thinning and retreat can reduce slope stability by diminishing the mechanical support of steep valley walls (debuttressing) and initiating stress-release fractures [7,44,45]. We observe that landslides are migrating upslope, following the retreating glaciers and permafrost degradation to ever higher elevations (Fig. 6b, c). (ii) Warming and degradation of permafrost reduce its rock-mechanical strength and weakens the shear resistance of ice-filled joints [46,47]. The loss of crack ice results in an increase in hydrologic connectivity of the rock mass, making it more sensitive to penetration of rainwater and meltwater. Such responses to warming have been demonstrated via field-measurements [43,44,48]. In the EHS, this sensitivity is heightened in degraded permafrost areas where the active layer has thickened on average by ∼8% since the 1960s [49]. (iii) The clustering of landslides on southward-facing hillslopes (Fig. 2d) reflects two ingredients: increased solar radiation during warm seasons intensifying freeze–thaw dynamics; and heavy precipitation falling on southward slopes via the Indian and East Asian summer monsoons [38]—especially if (iv) a rising proportion of the total precipitation falls as rain [50,51]. This shift is supported by a decrease in annual snowfall in the EHS of ∼40–80 mm/decade from 1979 to 2022 [40].

Precipitation exerts the strongest climatic influence on landslides in general, as shown by the regressions indicating that heavy precipitation events correlate with both larger and more frequent landslides (Fig. 3d–f), although the relationship with volume at high elevations is weak (Fig. 3f). The spatial distribution of landslides is also some function of precipitation, especially in years with extreme totals (for example, 1988, 1998, 2004, 2010, 2012, and 2020) (Fig. S3). Analyzing meteorological records (at Bomê and Nyingchi) from 1960 to 2003, Loibl *et al.* [38] note that precipitation and temperature are typically anti-correlated. Fewer landslides in the EHS occur at higher elevations (Fig. 2b) owing to the mostly solid precipitation and permafrost, which effectively limits landslide activity. Nevertheless, this situation may be changing in light of the growing number of anomalous years in which both higher precipitation and temperature coincide. These wetter and warmer years (1988, 1995, 1998, 2010, 2012, and 2020; Fig. S2) frequently hosted some of the largest total landslide volumes in our inventory (Fig. 3 and Fig. S9). Moreover, while the total volume of landslides shows no trend through time (Fig. 3a), the fraction contributed by high elevations increased sharply thanks to some very large landslides during four anomalously warm years, 2017–2020 (Figs S9 and S10). The influence of warming in the high mountains is, however, not the result of a suggested increase in the intensity and frequency of heavy rainfall [18,40,52]—no such trend is evident in the EHS record. As noted above, the most direct explanation for this elevation-dependent shift in landsliding behavior is glacier retreat, permafrost degradation, and an ongoing rise in the rain/snow ratio in the high mountains.

### Sediment mobilization and mountain denudation rates

The EHS landscape comprises vertically stacked erosional domains in which glacial-periglacial processes at high elevations transition to debris flows and fluvial processes lower down. Landslides are crucial to this sediment cascade and hillslopes coupled to fluvial erosion are the primary agents of mountain belt denudation [34,37]. Based on our 1987–2020 inventory (Table S2), we estimate that climate-related landslides mobilized on average 95.97 ± 29.20 million m^3^ of sediment per year which, expressed as volume displaced per unit area, gives a landslide mobilization rate of 1013 ± 308 m^3^ km^–2^ yr^–1^.

To gain a longer-timescale perspective on rates of climate-driven mass wasting in the EHS, the average landslide mobilization rate can also be expressed in terms of the denudation rate: 1.013 ± 0.308 mm yr^–1^. Notwithstanding that our data integrate just a few decades of observations, this denudation rate is of the same order as those of typical alpine glaciers ∼0.1–10 mm yr^–1^ when integrated over decades to millennia [53,54]. Moreover, sediment yields measured from alpine glaciers are likely to include a sizeable fraction of landslide-derived paraglacial debris [54], as testified by the extensive debris cover observed mantling high mountain glaciers [55]. Note for instance, the mass-wasting rate of ∼0.5–1.0 mm yr^–1^ estimated from ^10^Be measured in supraglacial debris on the Chhota Shigri Glacier, India Himalaya [56].

The striking 5-fold increase in the annual volume of landslides at high elevations in the EHS (Fig. 3c) demonstrates an elevation-dependence in landslide intensity and emphasizes the dynamism of hillslope response to recent climate change—seen also in the surging ratio of high/low elevation mobilization rates (Fig. S10). This leads us to suggest a similar dynamism may have characterized the dozens of climatic glacial-interglacial transitions during the Quaternary. Indeed, we argue that our EHS data may capture the essence of hillslope responses to deglaciation compressed into a matter of decades: warming drives glaciers to higher elevations, while permafrost degrades and landsliding intensifies as it follows in the path of the retreating glaciers.

Patterns of denudation in glaciated mountain belts are widely attributed to the negative feedback (dubbed the glacial buzzsaw) between topography and glacier mass-balance that concentrates glacial erosion within an elevation-band according to temperature and the dimensions of the glacier accumulation zone [57,58]. But what is the role of landsliding in the glacial buzzsaw? Their shared elevation-dependence and comparable erosional efficacy in high mountain belts suggest a relationship that warrants further scrutiny.

We constructed and analyzed a 34-year inventory of climate-related landslides in the EHS at annual resolution. Despite limitations in the inventory (see Methods), we find that global warming has intensified landsliding in the EHS and highlighted its importance to high mountain denudation. Future trends in precipitation in the EHS remain uncertain [59,60], but warming is expected to continue [60,61] and hence the intersections between glaciers, permafrost, and landslides will continue to expand rapidly with the likely result that landsliding will intensify further.

## MATERIALS AND METHODS

### Data

Landsat products provide the longest available continuous coverage of Earth’s surface with annual resolution. For the landslide mapping, we used Landsat 5 TM (1987–2011) and Landsat 8 OLI (2013–2020). Landsat 7 ETM+ lost its scan-line corrector, resulting in 22% loss of each scene after 2003 [62] and hence was used only for scenes not covered by Landsat 5 and 8 (2011 and part of 2012). Each time slice was set to October–February (a less cloudy period) and the post-imagery for one time slice seamlessly matched the pre-imagery for the next, thus ensuring continuous mapping. A constant resolution of 30 m was used.

Four topographic factors were considered for the landslide spatial distribution analysis: elevation, slope, aspect, and local relief—all derive directly from the ALOS Global Digital Surface Model (AW3D30) using the ArcGIS spatial toolbox. Lithology and fault data derive primarily from the dataset of 1:1.5 M geology map of China, while the Indian segment is digitized from the geology map of the Tibetan Plateau and its surrounding areas with the same scale (data from China Geological Survey https://geocloud.cgs.gov.cn/). We simplified the full range of lithologies into eleven groups based on their approximate relative rock strength (Fig. S5).

The extensive development of glaciers and permafrost at high elevations suggests an important influence of precipitation and temperature on geomorphic processes. Precipitation and air temperature data are sourced from the Third Pole Meteorological Forcing Dataset, which provides monthly, daily, and hourly data (1979–2022) at 1/30° resolution [63,64]. These data are generated by combining long-term ERA5 reanalysis with a short-term high-resolution atmospheric simulation and *in situ* observations—an approach that generally outperforms other reanalysis datasets [63,64]. Based on this dataset, the annual precipitation, heavy precipitation (that is, annual cumulative precipitation for days ≥ 25 mm), maximum daily precipitation, mean annual temperature, mean summer temperature, and maximum annual temperature in the EHS were calculated. We use a published map of permafrost distribution at 1 km scale [19], a dataset of permafrost changes at the same resolution spanning the 1960s to 2010s [49], and the Randolph Glacier Inventory (RGI) 7.0 data [65].

### Landslide inventory

Landslides were manually mapped by visually comparing pre- and post-event imagery for specific years. A polygon was delineated wherever a new bare patch appeared and the topography supported landslide occurrence (with a minimum of two adjacent pixels, >1800 m^2^). Human activities, such as deforestation, cultivation, engineering excavation and filling, etc., which may cause disturbances, were carefully excluded (Fig. S11). Bare patches gradually exposed due to glacier retreat have also been carefully excluded. Mapping was performed by an experienced mapper (first author) and revised following a review three months later. We believe that one expert maintained consistency in mapping standards as much as is possible, avoiding knowledge bias between different mappers. All mapped landslides encompass the areas of the scar, runout, and depositional zones. Given the difficulty with identifying landslide remobilization at 30 m resolution, only new landslides and major reactivations were recorded and treated as independent. Landslides in the EHS rarely occur outside the May–September monsoon season [66]. Nevertheless, where multiple images were available, those closer to the end of year were selected so as to minimize the issue of misattributing landslides into neighboring time slices.

Landslides in the EHS are typically triggered by rainfall, freeze–thaw cycles, glacier debuttressing, snow and ice meltwater, river erosion, earthquakes, sudden outburst floods, and human activities. The first five factors, being directly or indirectly influenced by climate factors, are categorized as climate-related, while the latter three factors are considered climate-independent. We set out to devise an inventory of landslides triggered exclusively by climate-related factors by eliminating those linked to seismicity, outburst flooding or human activities. During the period 1987–2020, the EHS experienced more than 200 earthquakes of M ≥ 4.0 with potential to trigger landslides [67–69], including five events of M ≥ 5.5. In accordance with the idea that for minor earthquakes the intensity of shaking depends on focal depth [69], we applied a 20 km focal depth limit for low-magnitude earthquakes (4.0 ≤ M < 5.5); a total of 88 earthquakes met this criterion. Coseismic landslides induced by the 2017 M 6.9 Mainling earthquake were removed directly following Hu *et al.* [70]. For other earthquakes, we applied the curves proposed by Keefer [67] that relate earthquake magnitude to the maximum distance of landslides from the epicenter. Any landslide occurring within the maximum distance was considered potentially coseismic (Fig. S12); a total of 177 landslides met this criterion.

In 2000, the giant Yigong landslide (∼3 × 10^8^ m^3^) formed a lake (∼30 × 10^8^ m^3^) that collapsed two months later [71,72] producing a major outburst flood. In the year that followed, 72 landslides were triggered in locations adjacent to the channel conveying the flood (Fig. S13). The Yigong landslide was influenced by long-term seismic activity and potentially triggered by an Ms 4.8 earthquake 13 km away [73,74]. Therefore, the Yigong landslide and all associated landslides were excluded from our inventory. Similarly, we excluded landslides induced by the Sedongpu outburst flood events in 2018 [75].

This remote region exhibits few landslides caused by human activities, and those that do occur are mostly associated with road construction. A landslide was attributed to road construction if its source area lies adjacent to the inner edge of a road and its path crosses the roadway (Fig. S11e). A total of 28 landslides were excluded on this basis.

### Size–frequency distribution of landslides

Landslide inventories are most commonly analyzed by plotting landslide probability density vs. size [76]. The probability density distribution of landslides, *p*(*A_L_*) can be defined as


(1) \begin{eqnarray*}
p\left( {{A}_L} \right) = \frac{1}{{{N}_{LT}}}\ \frac{{\delta {N}_L}}{{\delta {A}_L}},
\end{eqnarray*}

where *A_L_* is landslide area/volume, *N_LT_* is the total number of landslides in the inventory, $\delta {N}_L$ is the number of landslides with areas/volumes between *A_L_* and *A_L_+δ_AL_*, and *δA_L_* is the bin width [77]. Numerous observations show that a power-law is generally valid for the probability density distribution of large landslides and can be expressed as


(2) \begin{eqnarray*}
p\left( {{A}_L} \right) = c\ A_L^{ - \beta },
\end{eqnarray*}

where *c* is a normalization constant, and *β* is the power-law exponent. Small landslides typically diverge from the power-law due to undercounting [78]. The low resolution of the Landsat imagery used in our study means that some small landslides were inevitably missed, hence we focus on the distribution of large landslides.

### Conversion of landslide area to volume

A power-law scaling relationship [79] was used to estimate landslide volume from area


(3) \begin{eqnarray*}
V = \alpha \cdot {A}^\gamma ,
\end{eqnarray*}

where *V* is volume (m^3^), *A* is area (m^2^), and *α* (m^(3–2^*^γ^*^)^) and *γ* are scaling constants. To assess the sensitivity of landslide volumes to differing scaling constants, we follow the approach of Larsen and Montgomery [80] who apply three sets of scaling constants to characterize landslides in the eastern Himalaya (Table S4). We apply the scaling constants ‘group 1’, which are most appropriate for mixed soil-bedrock landslides. The results of applying scaling constants ‘group 2’ and ‘group 3’ are presented in the Supplementary Materials for comparison (Fig. S14 and Table S2).

The uncertainties associated with converting landslide areas to volumes stem mainly from the scaling constants (σ*_α_* and σ*_γ_*) and mapping errors. Following similar studies [81–83], we apply an arbitrary standard deviation of 20% of the landslide area to account for any potential mapping errors. These uncertainties are treated as independent variables and propagated into our volume estimates using a Gaussian distribution, yielding an average error of 29.7% in single-landslide volume estimates. For the standard deviation of the total landslide volume for a time period or region, we adopt a conservative approach by directly summing up the error of each landslide. In contrast, common methods assume independent volumes and ignore potential covariance [81,83]. This can underestimate total uncertainty when a few large landslides dominate, as their volume uncertainties are less likely to cancel out [81,83].

The average annual volume of hillslope material displaced per unit area, known as the landslide mobilization rate [84], is calculated by dividing the total landslide volume (Table S2) by the measured area and observation period (that is, (3263.02 ± 992.71) × 10^6^ m^3^/94 706 km^2^/34 yr = 1013.36 ± 308.30 m^3^ km^–2^ yr^–1^). By rearranging the units, landslide mobilization rate can also be expressed as the landscape denudation rate (that is, 1.013 ± 0.308 mm yr^–1^).

### Limitations

Studying the impact of climate change on landslides is reliant on long-term systematic inventories [31]. The primary approach employs satellite images, with their extensive historical archives; however, only Landsat can provide archives spanning more than 20 years [27–29,85]. We employed Landsat images for our 1987–2020 landslide inventory, acknowledging the following shortcomings: (i) data gaps. We relied predominantly on Landsat 5 TM (1987–2011) and Landsat 8 OLI (2013–2020). Landsat 7 images (22% data loss) were utilized for landslide mapping only for 2011 and part of 2012. Landslides occurring within the missing data bands have been ignored. A few studies have identified seasonal variations in landslide occurrence [86]; however, we could not confirm the season/month of landslide occurrence owing to the extensive cloud cover, which limits the number of useful images. (ii) Image resolution. To maintain consistency in the accuracy of landslide mapping, we used a standard image resolution of 30 m, which means that small landslides (<1800 m^2^) are likely to be missed. The neglect of small landslides in our inventory may potentially explain the lack of statistical significance regarding the size and frequency of landslides at low and medium elevations. Low-resolution image leads to ambiguous landslide boundary delineation, thereby introducing uncertainties in derived landslide area measurements. An assumed standard deviation of 20% was applied to all mapped features to accommodate any mapping error. (iii) Image quality. Poor image quality hinders the classification of landslide types during the mapping process. Similarly, magnitude and frequency changes of different landslide types are difficult to identify. Moreover, sensor improvements from Landsat 5 TM to Landsat 8 OLI may have enhanced detection of small landslides. However, a minimum area threshold of 1800 m^2^ helps limit this effect. No significant increase in landslide frequency has been observed since 2013, indicating limited impact of sensor improvements. Therefore, our observation on the increase in large landslides at high elevations remains robust. The growth of high-resolution satellite image archives, such as Planet (3 m resolution), means that long-term, high-resolution landslide inventories will become available in the future.

## Supplementary Material

nwag238_Supplemental_File

## Data Availability

The landslide inventory produced by this study is available in Zenodo (https://zenodo.org/records/14977406). The Landsat images can be downloaded from United States Geological Survey (USGS) website (https://earthexplorer.usgs.gov/). Earthquake data is also sourced from USGS (https://www.usgs.gov/programs/earthquake-hazards). The high-resolution near-surface meteorological forcing dataset for the Third Pole region can be downloaded from National Tibetan Plateau Data Center (https://data.tpdc.ac.cn/zh-hans/data/44a449ce-e660-44c3-bbf2-31ef7d716ec7). The dataset of 1:1.5 M geology map of China, and the 1:1.5 M geology map of the Tibetan Plateau and its surrounding areas can be downloaded upon request from the China Geological Survey website (https://geocloud.cgs.gov.cn/). The ALOS Global Digital Surface Model (AW3D30) can be downloaded at https://www.eorc.jaxa.jp/ALOS/en/dataset/aw3d30/aw3d30_e.htm. The map of permafrost distribution comes from the Supplementary of Zou *et al.* [19] (https://doi.org/10.5194/tc-11-2527-2017-supplement). The dataset of permafrost changes spanning 1960s to 2010s can be downloaded from National Tibetan Plateau Data Center (https://data.tpdc.ac.cn/zh-hans/data/ade493c8-3692-4871-bcb4-a4fabaef04a9). RGI 7.0 data can be download at http://www.glims.org/RGI/.
