# Supplementary material for: Elevation-dependent landsliding driven by climate change in the eastern Himalayan syntaxis
Source: Natl Sci Rev. 2026 Apr 24;13(10):nwag238. doi: 10.1093/nsr/nwag238 (PMC13273571; doi:10.1093/nsr/nwag238)
Supplement: nwag238_Supplemental_File [file nwag238_Supplemental_File.pdf]

Supplementary Materials

Elevation-dependent landsliding driven by climate change in the eastern Himalayan syntaxis

Chengbin Zou[1,†], John D. Jansen[2,†], Xiangyang Dou[1,3], Lanxin Dai[1], Qiang Xu[1], Xuanmei Fan[1,*,†]

[1] State Key Laboratory of Geohazard Prevention and Geoenvironment Protection, Chengdu University of Technology, Chengdu 610059, China.

[2] GFÚ Institute of Geophysics, Czech Academy of Sciences, 141 00 Prague 4, Czechia.

[3] Faculty of Geo-Information Science and Earth Observation, University of Twente, 7522 NH, Enschede, the Netherlands.

* Corresponding author: Xuanmei Fan (fxm_cdut@qq.com)

[†] These authors contributed equally to this work

This PDF file includes:

Figures S1 to S15
Tables S1 to S4
References

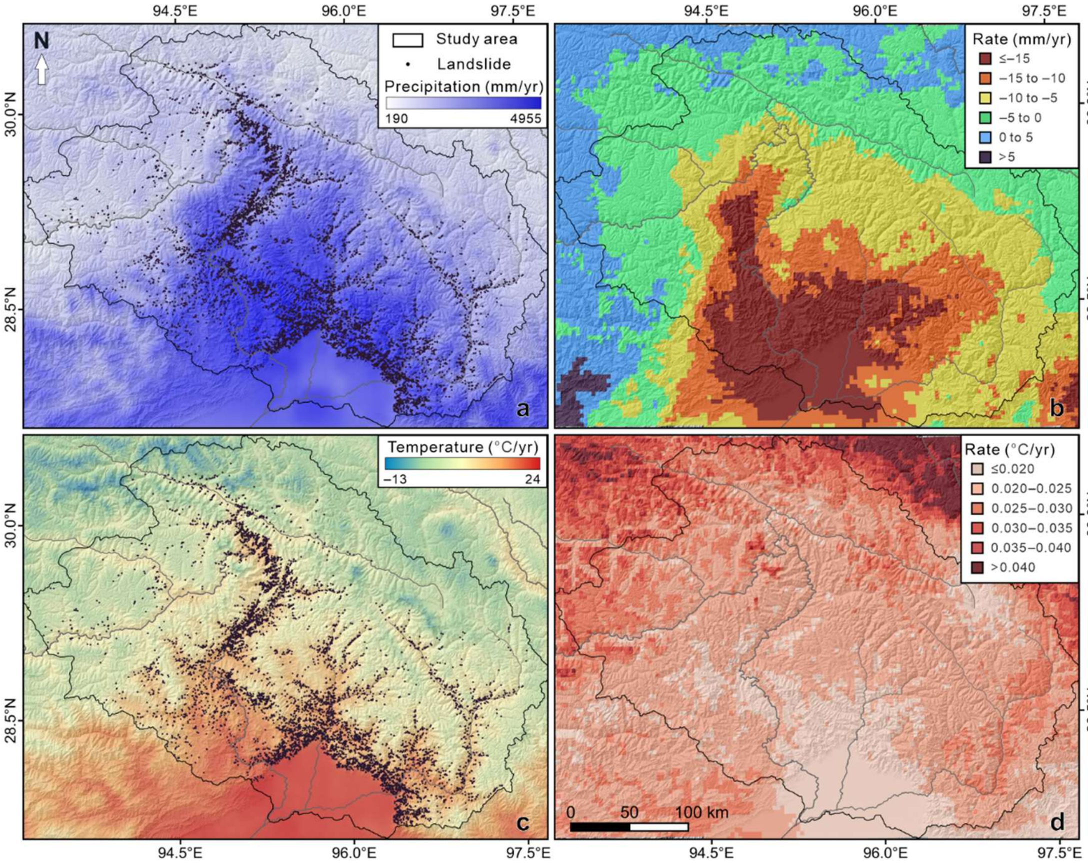

Fig. S1. Spatial patterns of landslides, precipitation and temperature (1979–2022). (a) Mean annual precipitation; change rate of mean annual precipitation; (c) mean annual temperature; (d) change rate of mean annual temperature. These data are from Jiang et al. [1,2]

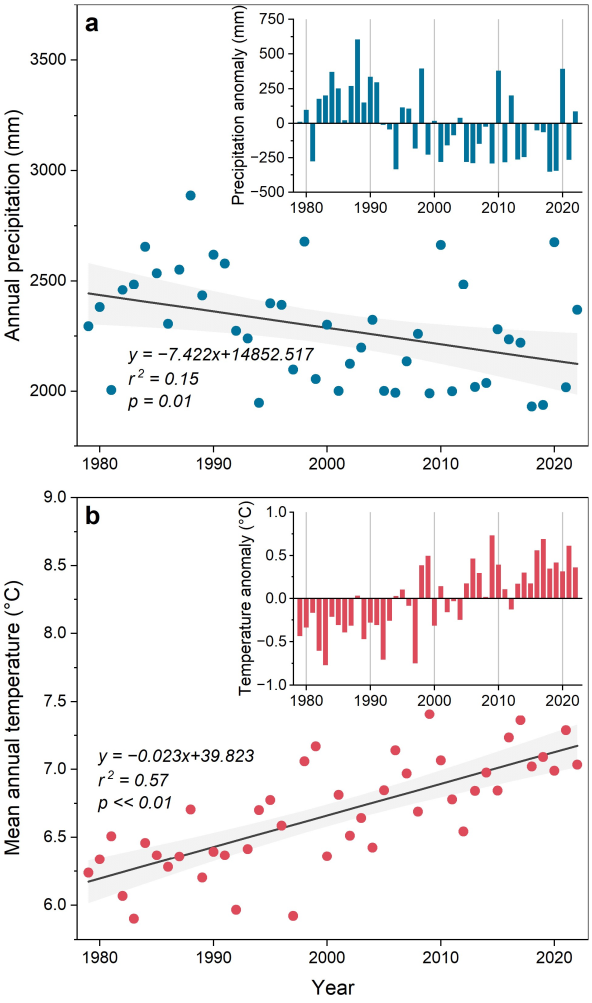

Fig. S2. Historical trends (1979–2022) in precipitation and temperature. (a) Annual precipitation fitted with a linear regression and 95% confidence intervals. Inset shows the same data as a histogram of precipitation anomaly (deviation from mean annual precipitation). (b) Mean temperature fitted with a linear regression and 95% confidence intervals, showing a warming rate of 0.23 ± 0.03 °C decade–1. Inset shows the same data as a histogram of temperature anomaly (deviation from mean annual temperature). These data represent the regional averages from our study area, calculated from the Third Pole Meteorological Forcing Dataset [1,2].

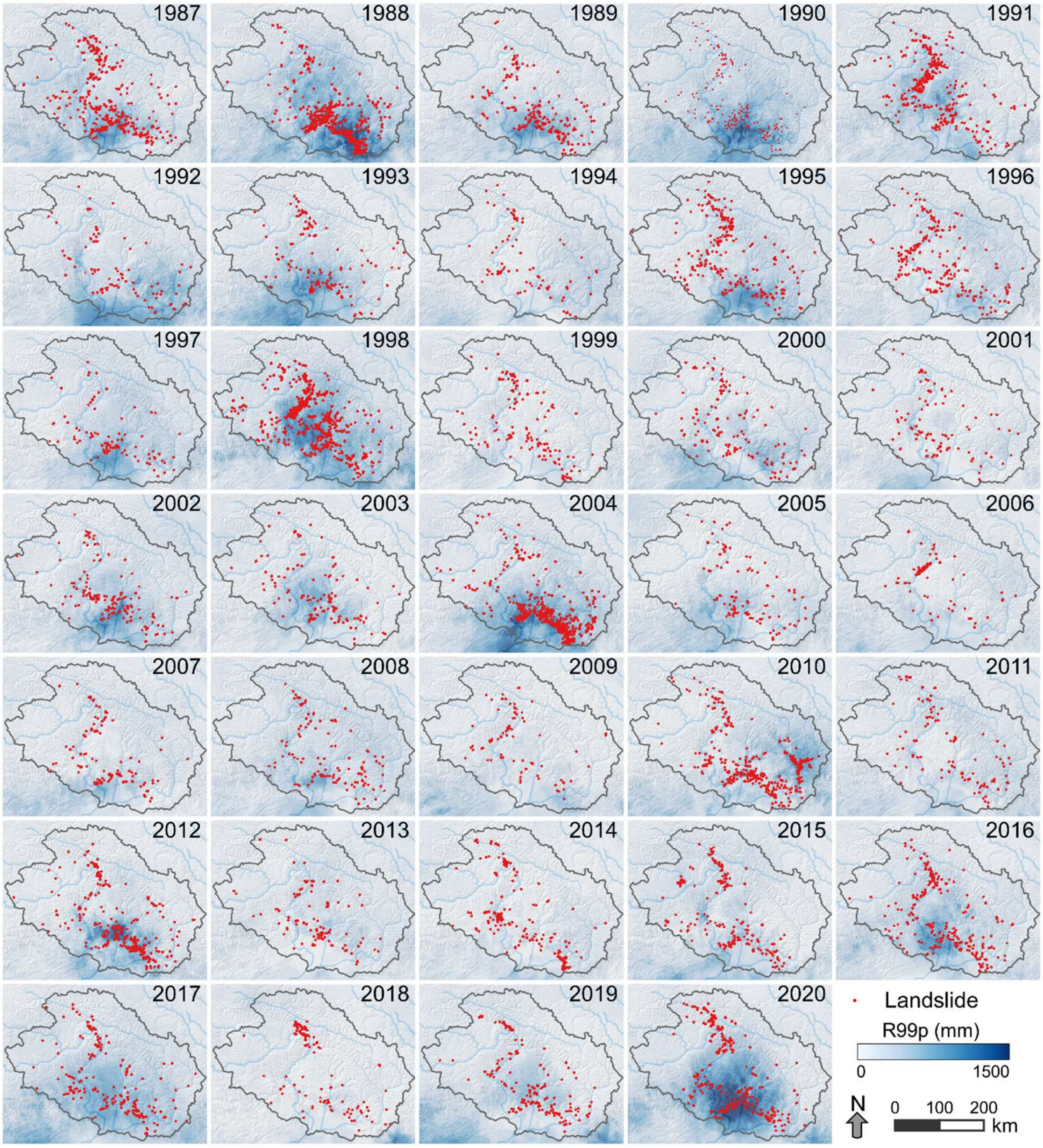

Fig. S3 Annual distribution of landslides and 99th percentile extreme precipitation (R99p).

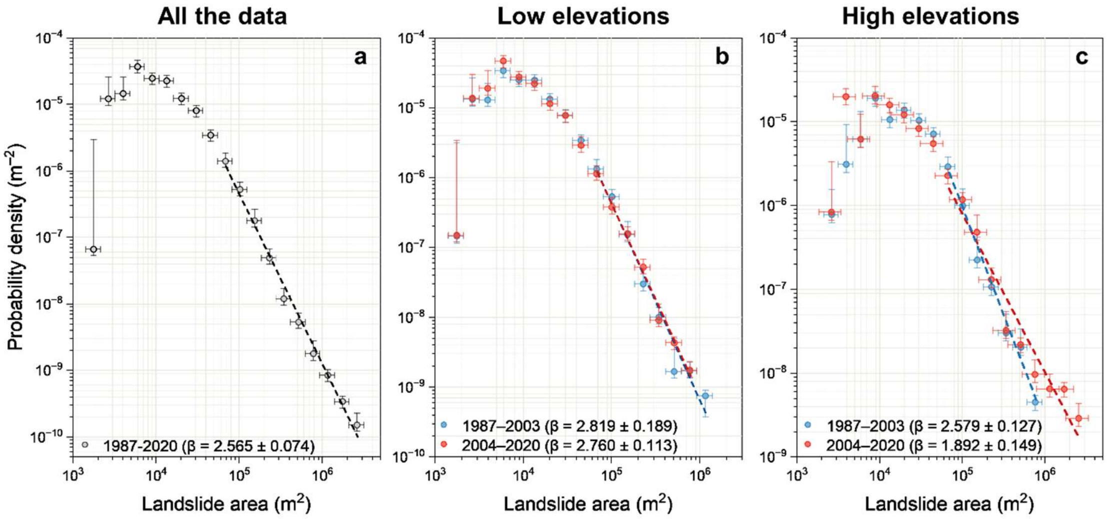

Fig. S4. Landslide are-frequency distributions. (a) Area-frequency distribution for all landslides (1987–2020); those with area >50,000 m^2 follow a power-law distribution [3,4]. (b) Comparison of low-elevation (< 3000 masl) landslide area distributions in split time-intervals (1987–2003 and 2004–2020); the two periods follow the same power distribution, indicating no change. (c) Comparison of high elevation (> 3000 masl) landslide area distributions for the same intervals (1987–2003 and 2004–2020).

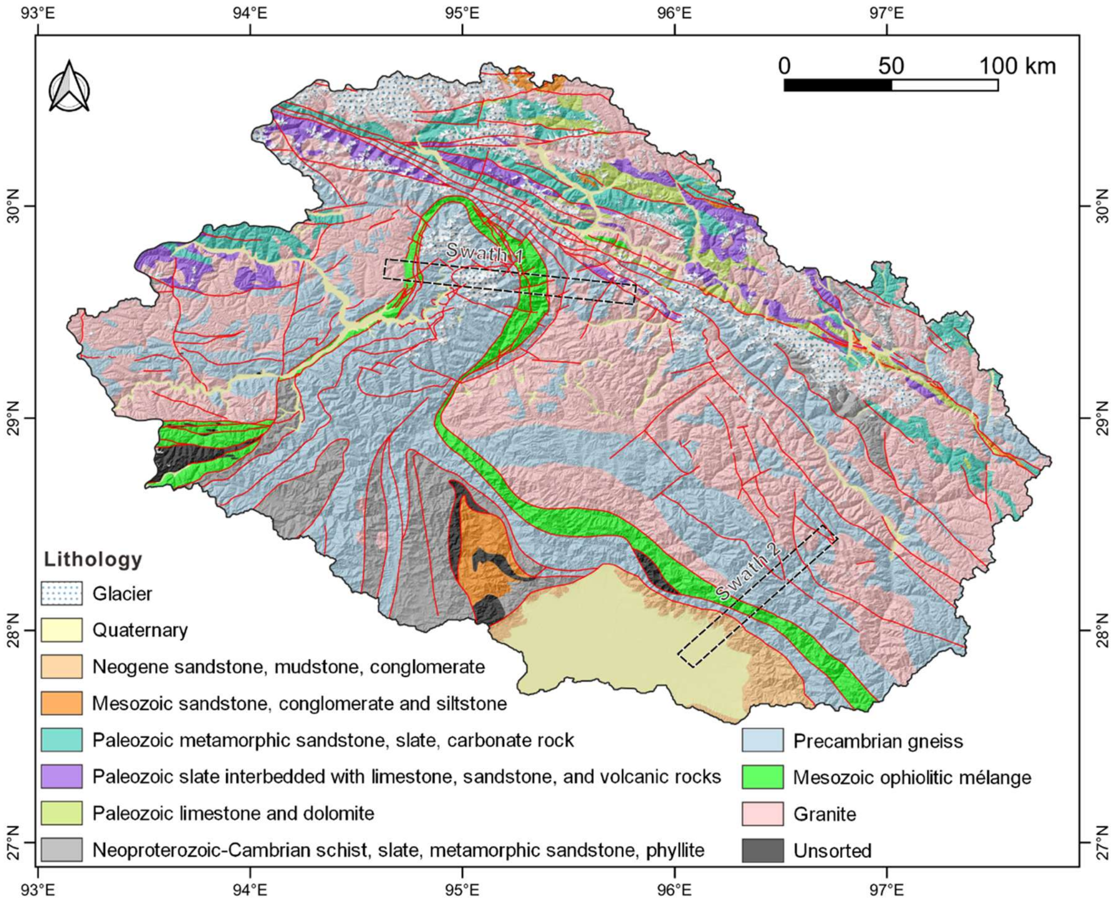

Fig. S5. Simplified lithological map. Lithology and fault data derive primarily from the geology map of China (1:1.5 M) with the Indian segment digitised from the geology map of the Tibetan Plateau and its surrounding areas (data accessed from the China Geological Survey, https://geocloud.cgs.gov.cn/). Strata with similar lithologies were merged and simplified into a total of eleven groups. Swath profiles (Fig. 5) indicated by black dashed boxes.

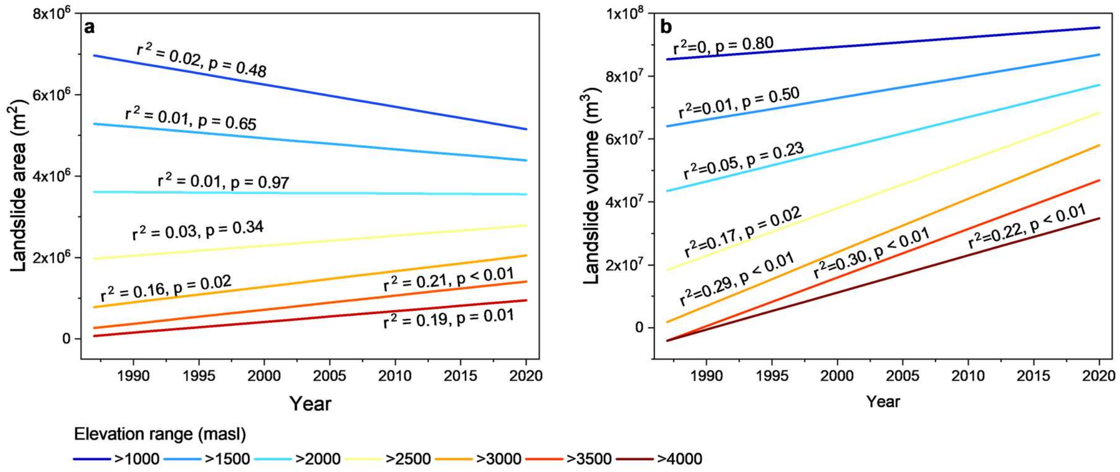

Fig. S6. Historical trends (1987–2020) in landslide area and volumes for landslides above threshold elevations. These data show that elevations >3000 masl have the strongest trends (steepest slope and lowest *p*) for enhanced landslide area and volumes over time. Landslide volumes were estimated using the first group of scaling constants. These regressions are based on non-transformed data.

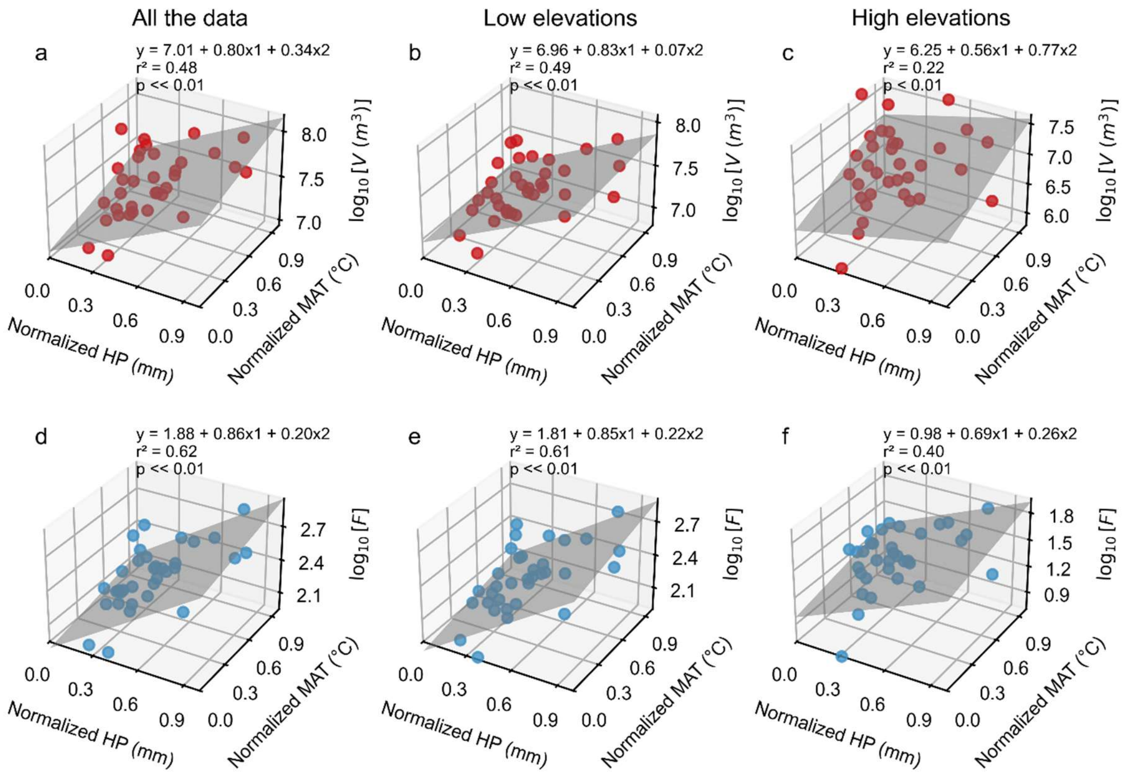

Fig. S7. Binary regression relationships between landslide volume (V) and frequency (F) versus climate factors at low and high elevations. (a–c) Landslide volume versus heavy precipitation, HP (annual cumulative precipitation counting only days ≥ 25 mm) and mean annual temperature, MAT. (d–f) Landslide frequency versus HP and MAT. Note that volume and frequency data are transformed (log10) to achieve more constant residual variance. Note that binary regression yields stronger relationships between volume, frequency and climate than simple linear regression presented in Figure 3.

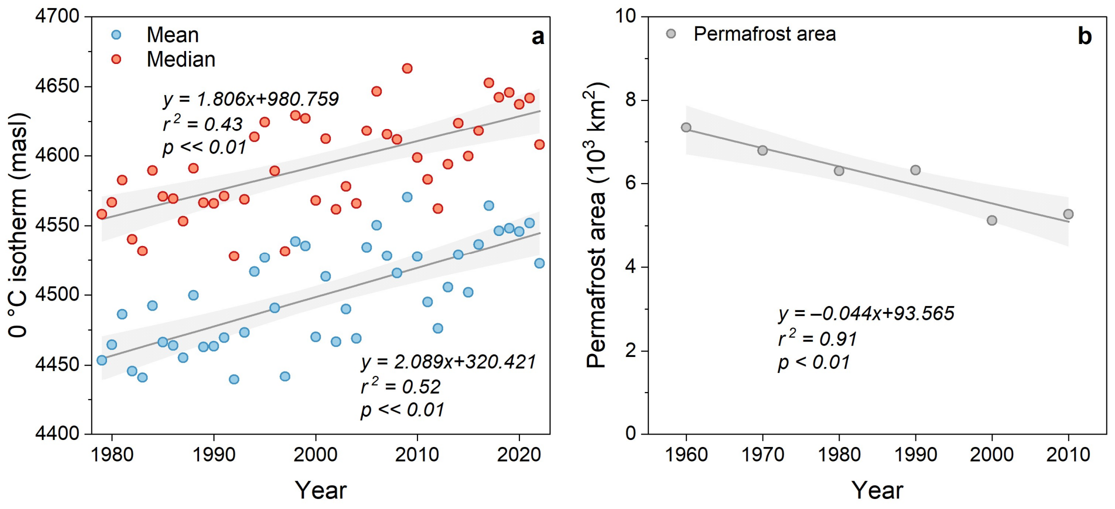

Fig. S8. Historical trends in the elevation of the 0 °C isotherm and area of permafrost. (a) Elevation-change in the 0 °C isotherm (1979–2022) generated from the mean temperature data of Jiang et al. [1]. Mean and median elevation of the 0 °C isotherm is extracted from the ALOS Global Digital Surface Model (AW3D30). (b) Reduction in permafrost area of ~ 30 % (1960s–2010s) based on data from Ran et al. [5] with spatial resolution of 1 km and temporal resolution of 10 years.

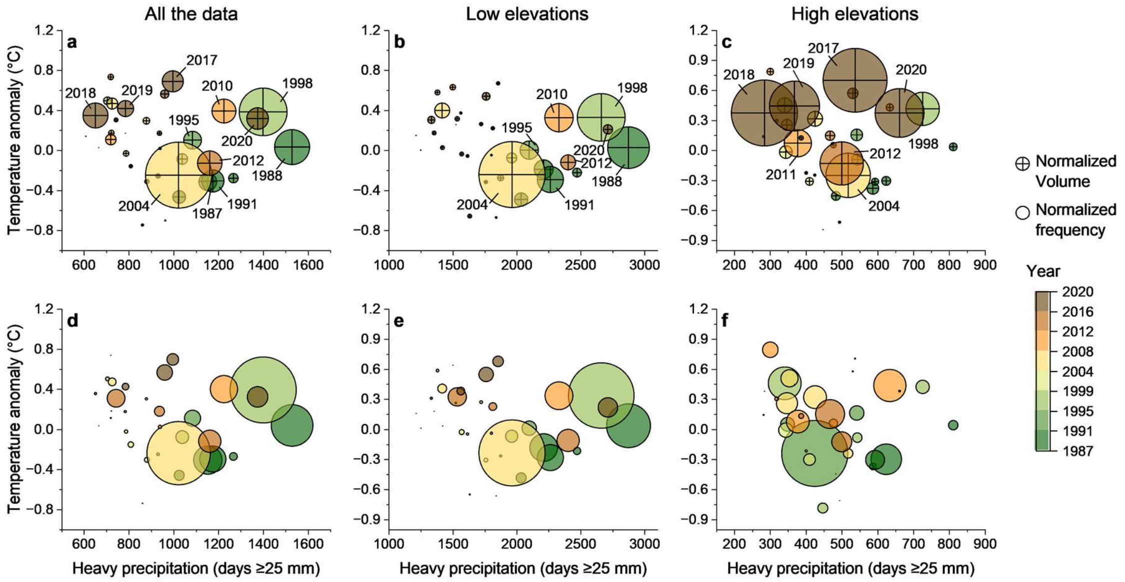

Fig.S9. Relationship between temperature anomaly and precipitation at low and high elevations. Temperature anomaly (deviation from mean annual temperature) versus heavy precipitation (annual cumulative precipitation for days ≥ 25 mm). Size of filled circles is scaled to indicate normalised landslide volumes/frequency per year.

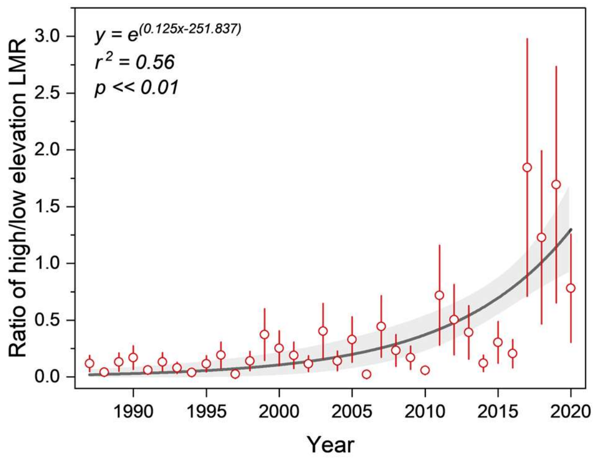

Fig. S10. Historical changes in the ratio of high / low elevation LMR (landslide mobilization rate): the average annual volume of hillslope material displaced per unit area (m^3 km^{-2} yr^{-1}). Note some very large landslides occurred during four anomalously warm years, 2017–2020. Data are fitted with an exponential regression and 95% confidence intervals.

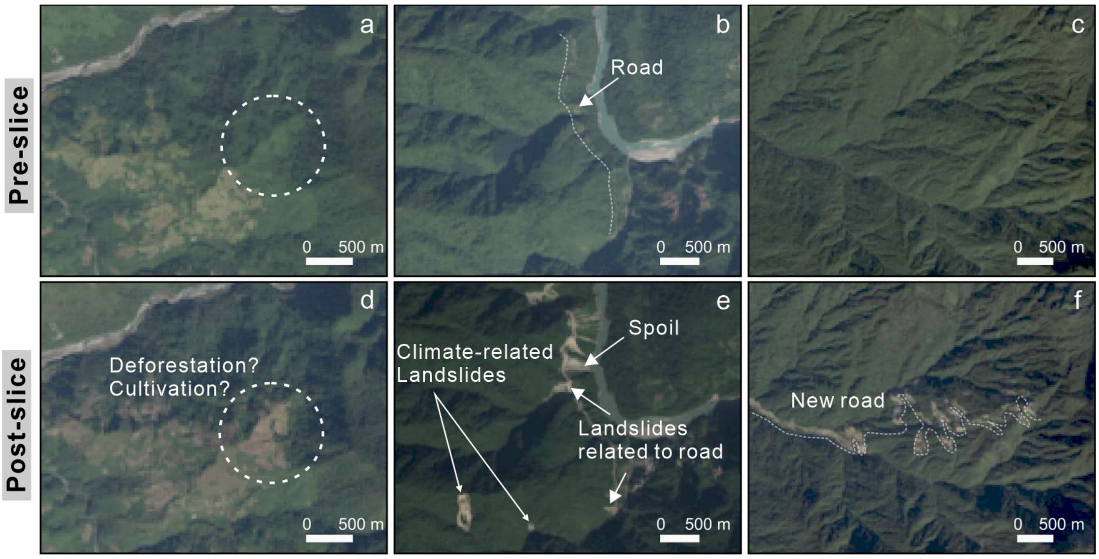

Fig. S11. Some examples show the disturbances caused by human activities. These human activities include deforestation, cultivation and engineering excavation and filling, etc., which were carefully excluded during the landslide mapping. While their identification is challenging, the surrounding topography and land use can provide useful clues. The direction of the roads is marked with an offset white dashed line to help understand.

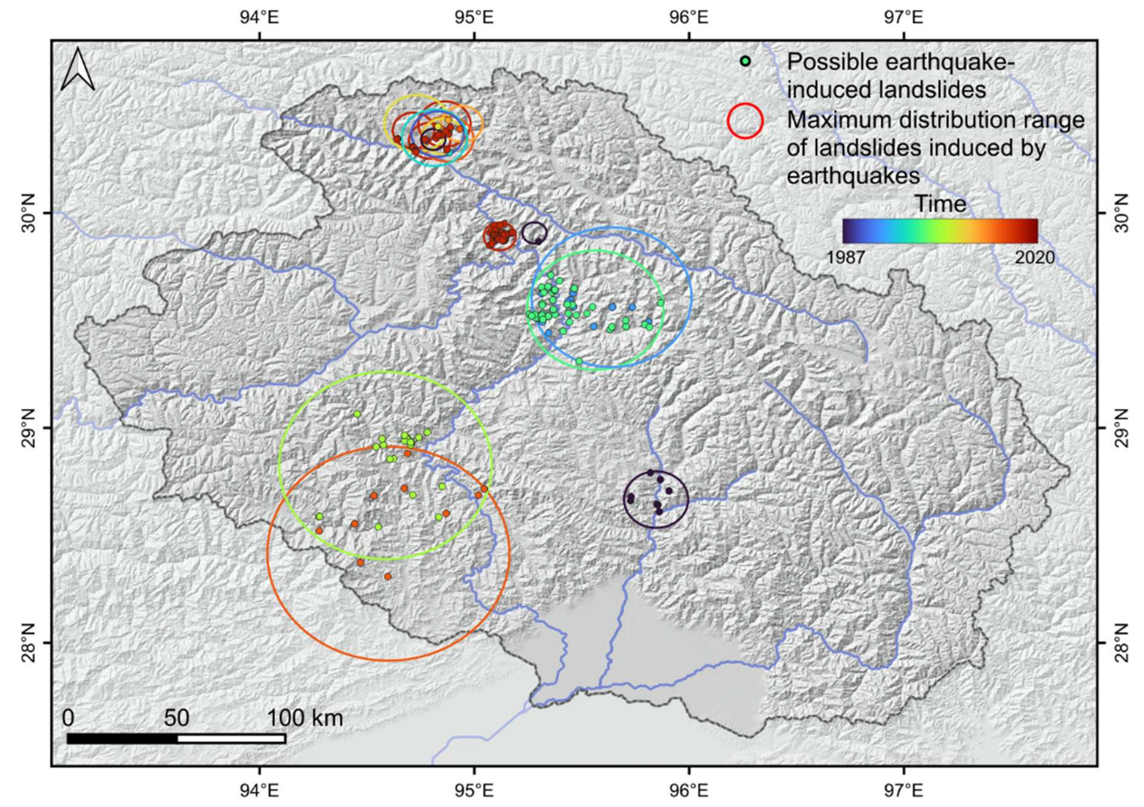

Fig. S12. Earthquake-triggered landslides excluded from our inventory. The potential maximum distribution range of landslides induced by earthquakes was generated based on curves proposed by Keefer [6]. A total of 177 landslides linked to 17 earthquakes were identified and excluded from our inventory. Also excluded were landslides induced by the 2017 Ms 6.9 Mainling earthquake based on the data from Hu et al. [7] (not displayed here).

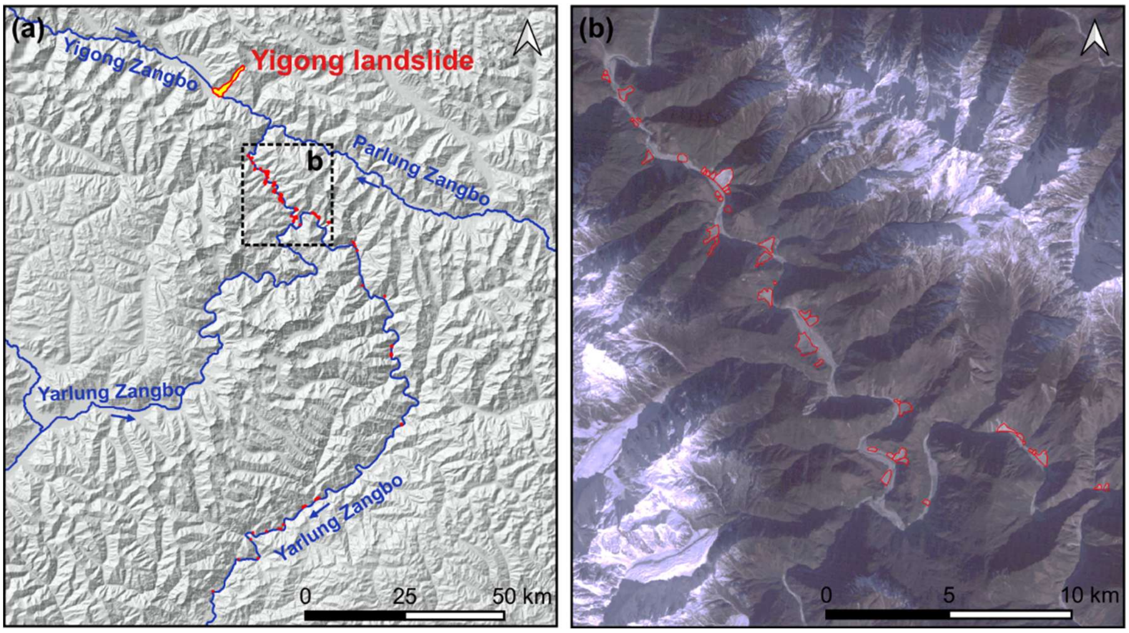

Fig. S13. Landslides triggered by the 2000 outburst flood from Yigong landslide-dammed lake. (a) Distribution of all 72 landslides (red) induced by the outburst flood. All were excluded from our inventory. (b) Enlarged image (Landsat 5, December 22, 2000) showing details of landslide (red outlines).

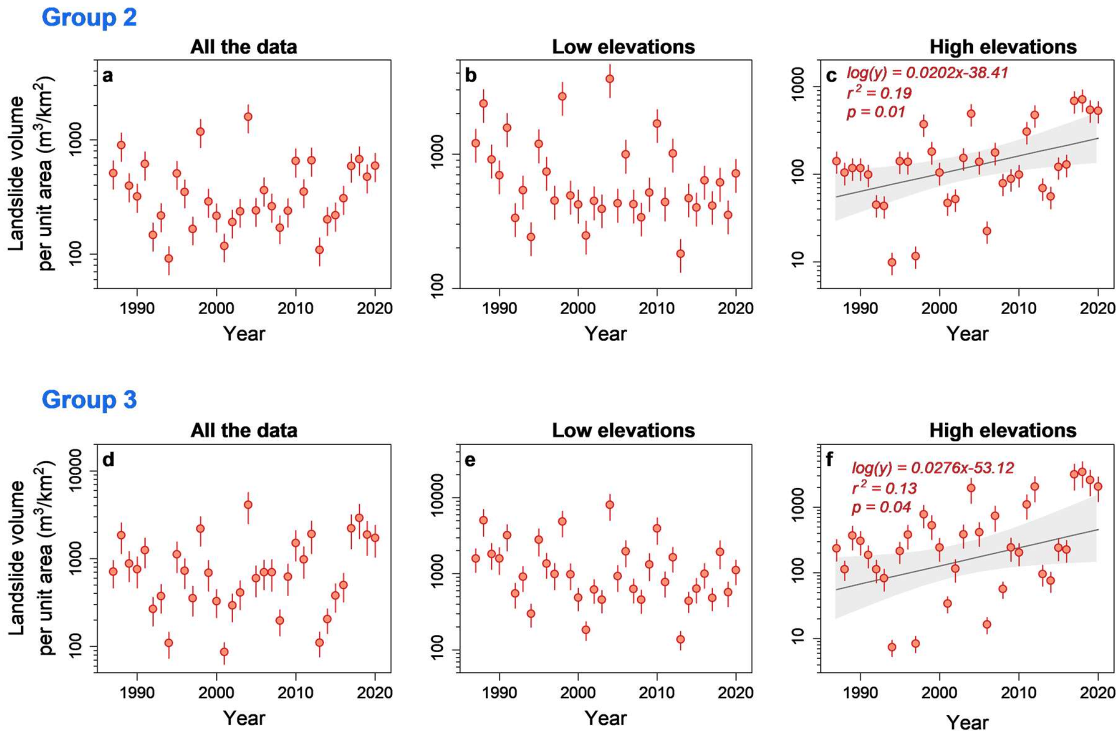

Fig. S14. The trends of estimated landslide volumes based on the different scaling constants. Group 2: $\gamma = 1.332 \pm 0.005$, $\log_{10}\alpha = -\,0.836 \pm 0.015$); Group 3: for landslide area < 10^5, $\gamma = 1.262 \pm 0.009$, $\log_{10}\alpha = -\,0.649 \pm 0.021$; for landslide area > 10^5, $\gamma = 1.41 \pm 0.02$, $\log_{10}\alpha = -\,0.63 \pm 0.06$). Please refer to Figure 3a-c for the results of Group 1. Notably, the trends of annual landslide volume are largely consistent across different sets of scaling constants.

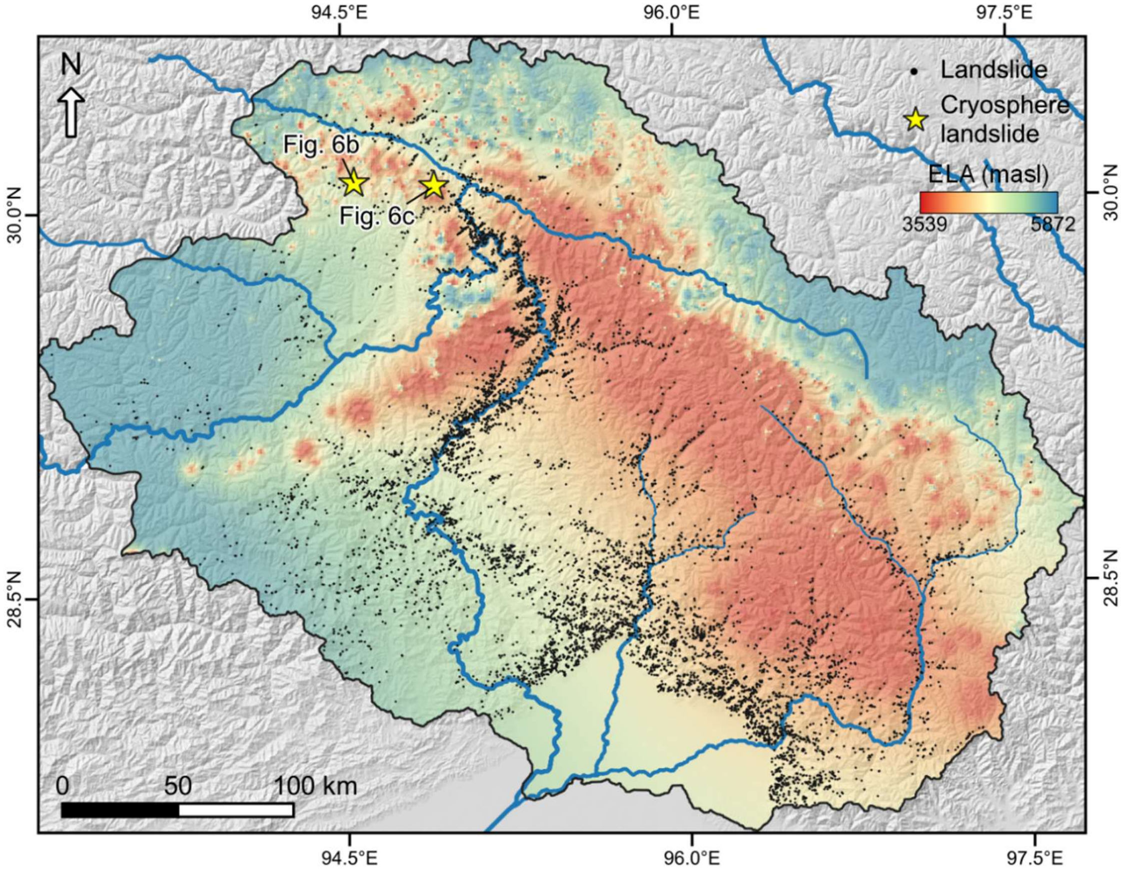

Fig. S15. Map of glacier Equilibrium Line Altitude (ELA), the perennial snowline. ELA is approximated by the median elevation of each glacier [8] which was extracted based on the ALOS Global Digital Surface Model and the Randolph Glacier Inventory [9]. The map was generated by interpolating glacier polygon geometric centre points in and around the study area based on inverse distance weight interpolation. Yellow stars indicate the locations of landslides shown in Figure 6b,c.

Table S1. Number and proportion of landslides per unit area.

Landslide area (m^2)	Number	Proportion (%)
medium (1800 ~ 10^4)	3043	35.4
large (10^4 ~ 10^5)	5296	61.5
very large (10^5 ~ 10^6)	263	3.1
giant (> 10^6)	6	0.07

Table S2. Landslide inventory data (1987–2020). Total study area is 94,706 km^2.

Year	Number	Area (km^2)	Volume ± uncertainty (10^6 m^3)[a]		
			$\gamma = 1.360 \pm 0.010$; $\log_{10}\alpha = -0.590 \pm 0.030$	$\gamma = 1.332 \pm 0.005$; $\log_{10}\alpha = -0.836 \pm 0.015$	Area<10^5 m^2, $\gamma = 1.262 \pm 0.009$, $\log10\alpha = -0.649 \pm 0.021$ Area>10^5 m^2, $\gamma = 1.410 \pm 0.020$, $\log10\alpha = -0.630 \pm 0.060$
1987	431	9.76	115.51 ± 34.79	48.45 ± 13.28	67.13 ± 23.14
1988	588	14.93	205.83 ± 62.47	84.94 ± 23.33	174.70 ± 65.95
1989	216	6.58	90.48 ± 27.39	37.50 ± 10.29	83.03 ± 31.09
1990	192	5.15	73.63 ± 22.36	30.32 ± 8.33	71.68 ± 27.39
1991	408	10.50	140.20 ± 42.44	58.15 ± 15.96	118.42 ± 44.11
1992	98	2.64	33.22 ± 10.02	13.88 ± 3.80	25.29 ± 9.15
1993	135	3.89	49.08 ± 14.80	20.51 ± 5.62	35.45 ± 12.70
1994	88	1.83	20.44 ± 6.14	8.62 ± 2.36	10.36 ± 3.42
1995	292	8.15	116.65 ± 35.43	48.04 ± 13.20	106.20 ± 40.38
1996	248	6.18	79.41 ± 23.99	33.06 ± 9.07	68.74 ± 25.49
1997	108	2.86	37.75 ± 11.42	15.68 ± 4.30	33.43 ± 12.46
1998	875	20.96	268.84 ± 81.26	111.88 ± 30.69	208.67 ± 76.60
1999	144	4.49	66.51 ± 20.20	27.38 ± 7.52	65.30 ± 24.86
2000	160	3.95	48.80 ± 14.72	20.40 ± 5.59	31.06 ± 10.93
2001	104	2.37	26.29 ± 7.89	11.11 ± 3.04	8.18 ± 2.24
2002	163	3.60	42.93 ± 12.93	18.01 ± 4.93	27.72 ± 9.70
2003	137	4.12	53.64 ± 16.20	22.35 ± 6.13	38.86 ± 14.00
2004	842	24.08	368.21 ± 112.21	150.59 ± 41.41	387.97 ± 150.83
2005	108	3.78	55.27 ± 16.77	22.79 ± 6.26	56.50 ± 21.49
2006	191	6.17	82.64 ± 24.98	34.36 ± 9.43	66.54 ± 24.37
2007	127	3.91	60.83 ± 18.54	24.87 ± 6.84	66.13 ± 25.73
2008	139	3.31	38.10 ± 11.45	16.05 ± 4.39	18.62 ± 6.07
2009	97	3.57	55.17 ± 16.79	22.62 ± 6.22	58.94 ± 22.72
2010	420	11.17	148.84 ± 45.03	61.80 ± 16.96	143.31 ± 53.87
2011	114	4.81	81.84 ± 24.98	33.31 ± 9.17	93.15 ± 36.33
2012	361	9.24	155.17 ± 47.56	62.74 ± 17.28	181.23 ± 72.19
2013	124	2.24	24.32 ± 7.30	10.28 ± 2.81	10.48 ± 3.28
2014	310	4.53	44.54 ± 13.32	18.98 ± 5.19	19.35 ± 6.00
2015	221	4.27	49.33 ± 14.84	20.73 ± 5.68	36.14 ± 12.91
2016	280	5.86	69.34 ± 20.88	29.10 ± 7.97	47.32 ± 16.73
2017	235	6.18	142.36 ± 44.30	55.81 ± 15.44	210.52 ± 87.78
2018	126	5.70	165.39 ± 51.64	64.24 ± 17.80	276.23 ± 115.47
2019	182	4.88	114.43 ± 35.57	44.92 ± 12.43	177.69 ± 73.74
2020	343	8.69	138.05 ± 42.12	56.30 ± 15.49	162.86 ± 63.81
Total	8607	224.4	3263.02 ± 992.71	1339.77 ± 368.20	3187.20 ±1226.92
Mean	253	6.6	95.97 ± 29.20	39.40 ± 10.83	93.74 ± 36.09
landslide mobilization rate (m^3 km^{-2} yr^{-1})			1013.36 ± 308.30	416.08 ± 114.35	989.81 ± 381.03

Table S3. Correlation analysis between different meteorological indicators and landslide volume (V) and frequency (F). Note that volume and frequency data are transformed (log_{10}) to improve the correlation. All meteorological data are the average values of the study area.

Meteorological indicators		All data		Low elevation		High elevation	
		Log[V]	Log[F]	Log[V]	Log[F]	Log[V]	Log[F]
Precipitation (mm)	Annual precipitation	0.57**	0.75**	0.71**	0.72**	0.17	0.66**
	Heavy precipitation	0.62**	0.77**	0.70**	0.76**	0.23	0.59**
	Maximum daily precipitation	0.28	0.38*	0.32	0.31	0.06	0.33
Temperature (°C)	Mean annual temperature	0.17	0.04	0.09	0.02	0.36*	0.14
	Mean summer temperature	0.01	–0.20	–0.36*	–0.28	0.28	–0.14
	Maximum annual temperature	–0.17	–0.17	–0.48**	–0.31	–0.07	–0.18

*p<0.05, **p<0.01.

Table S4. Landslide volume to area scaling constants used in this study [10]

Group.	γ	$\log_{10}\alpha$	Applicable landslide types and regions
1	1.36 ± 0.01	– 0.59 ± 0.03	Mixed soil and bedrock landslides (Himalaya)
2	1.332 ± 0.005	– 0.836 ± 0.015	All landslides (global)
3[a]	1.262 ± 0.009	– 0.649 ± 0.021	Landslide area <10^5 m^2 (global)
	1.41 ± 0.02	– 0.63 ± 0.06	Landslide area >10^5 m^2 (global)

[a] Based on the assumption that landslides with areas <10^5 m^2 comprise predominantly soil and those with areas >10^5 m^2 comprise predominantly bedrock.

References

1. Y. Jiang, W. Tang, K. Yang, et al. Development of a high-resolution near-surface meteorological forcing dataset for the third pole region. *Sci. China Earth Sci.* 2025; **68**: 1274-1290.
2. Y. Jiang, K. Yang, Y. Qi, et al. TPHiPr: a long-term (1979–2020) high-accuracy precipitation dataset (1⁄30°, daily) for the third pole region based on high-resolution atmospheric modeling and dense observations. *Earth Syst. Sci. Data* 2023; **15**: 621-638.
3. C. P. Stark, N. Hovius. The characterization of landslide size distributions. *Geophys. Res. Lett.* 2001; **28**: 1091-1094.
4. H. Tanyas, C. J. van Westen, K. E. Allstadt, et al. Factors controlling landslide frequency-area distributions. *Earth Surf. Process. Landf.* 2019; **44**: 900-917.
5. Y. Ran, X. Li, T. Che, et al. Current state and past changes in frozen ground at the third pole: a research synthesis. *Adv. Clim. Chang. Res.* 2022; **13**: 632-641.
6. D. K. Keefer. Landslides caused by earthquakes. *GSA Bulletin* 1984; **95**: 406-421.
7. K. Hu, X. Zhang, Y. You, et al. Landslides and dammed lakes triggered by the 2017 ms6.9 milin earthquake in the tsangpo gorge. *Landslides* 2019; **16**: 993-1001.
8. R. J. Braithwaite, S. C. B. Raper. Estimating equilibrium-line altitude (ELA) from glacier inventory data. *Ann. Glaciol.* 2009; **50**: 127-132.
9. RGI Consortium, Data from "Randolph glacier inventory - a dataset of global glacier outlines, version 7.0.". Deposited 2025-02-27 14:38:00.
10. I. J. Larsen, D. R. Montgomery, O. Korup. Landslide erosion controlled by hillslope material. *Nat. Geosci.* 2010; **3**: 247-251.
